# Supplementary material for: Integrated analysis of the aqueous humor microbiome and lens capsule transcriptome in high myopia cataract: a pilot study
Source: Front Med (Lausanne). 2026 Jun 16;13:1845205. doi: 10.3389/fmed.2026.1845205 (PMC13314463; doi:10.3389/fmed.2026.1845205)
Supplement: Supplementary file 3 [file Data_Sheet_1.zip › 3.Beta_Diversity/PCA/C372089/PCA_3D.pdf]

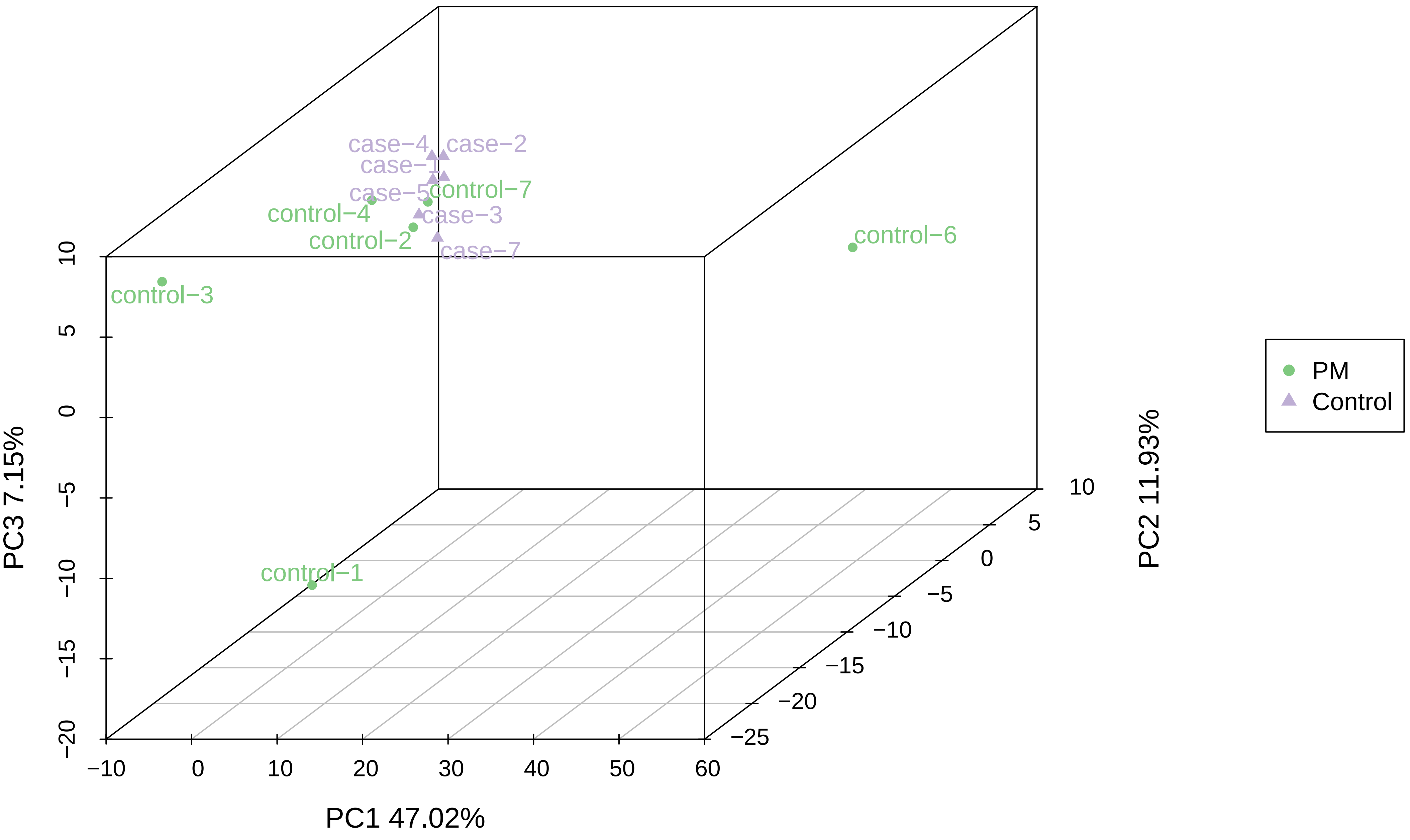
case−4
case−2
case−1
case−5
control−7
control−4
case−3
control−2
case−7
control−6
control−3
control−1
PM
Control
PC1 47.02%
PC2 11.93%
PC3 7.15%
−10
0
10
20
30
40
50
60
−25
−20
−15
−10
−5
0
5
10
